# Cross-cultural adaptation and initial evaluation of measurement properties of the Brazilian Portuguese version of the Lower Extremity Motor Function Test (LE-MFT)

**DOI:** 10.1055/s-0046-1827052

**Published:** 2026-08-13

**Authors:** Laura Hellen S. Cerqueira Gomes, Darlene Barbosa Marques, Lisandra Cristine de Faria, Gitendra Uswatte, Edward Taub, Sarah dos Anjos, Natalia Duarte Pereira

**Affiliations:** 1 Universidade Federal de São Carlos, Departamento de Fisioterapia, São Carlos SP, Brazil.; 2 University of Alabama at Birmingham, Department of Psychology, Birmingham AL, United States.; 3 University of Alabama at Birmingham, Department of Occupational Therapy, Birmingham AL, United States.

**Keywords:** Lower Extremity, Stroke, Self-Help Devices, Motor Activity, Validation Study

## Abstract

**Background:**

Lower extremity motor function assessments are essential to evaluate mobility and independence in stroke survivors. However, widely-used instruments do not account for the use of assistive devices, which may limit their sensitivity in detecting functional changes over time.

**Objective:**

To translate and cross-culturally adapt the Lower Extremity Motor Function Test (LE-MFT) into Brazilian Portuguese and to evaluate its initial measurement properties.

**Methods:**

The present study reports the translation, cross-cultural adaptation, evaluation of internal consistency, interrater reliability, construct validity, and floor/ceiling effects of the LE-MFT Brazil. The LE-MFT was translated and culturally adapted following international guidelines. Fifty stroke survivors were evaluated by two independent raters to examine the interrater reliability. Construct validity was assessed through correlations with the Timed Up and Go (TUG) test and the 10-Meter Walk Test (10MWT). Floor and ceiling effects were also analyzed.

**Results:**

All LE-MFT scales showed excellent interrater reliability (intraclass correlation coefficient [ICC] = 0.86–0.96) and internal consistency (Cronbach's Alpha [α] = 0.92–0.96). Strong correlations were found between the LE-MFT and TUG (r ≥ 0.78; *p* < 0.001), and between the LE-MFT and 10MWT (r ≥ 0.71; *p* < 0.001), supporting construct validity. No floor nor ceiling effects were observed, except on the Equipment scale, which showed a ceiling effect in 38% of the participants.

**Conclusion:**

The Brazilian version of the LE-MFT is a valid and reliable instrument to assess lower-limb motor function in individuals with chronic stroke. Its consideration of assistive-device use provides added clinical value for tracking functional independence.

## INTRODUCTION

Stroke is a leading cause of long-term disability, and it often results in persistent deficits in lower-extremity motor function, limiting mobility, independence, and participation in daily life. 1 2 3 4 As a result, evaluating lower-limb function in individuals with chronic stroke is essential to guide rehabilitation and monitor outcomes.

Several instruments are commonly used to assess mobility in this population, such as the Timed Up and Go (TUG) test, the 10-Meter Walk Test (10MWT), and the Fugl-Meyer Assessment of Lower Extremity. 5 6 7 While these measures are widely adopted, they primarily focus on gait speed, balance, or isolated motor impairments, and they may not capture the complexity of real-world performance or the quality of task execution. Furthermore, they do not assess the need for assistive devices or orthoses during functional tasks—factors that are highly relevant to determine functional independence. 8 9

The Lower Extremity Motor Function Test (LE-MFT) is an observational instrument designed to assess functional motor performance of the lower limb in adults with neurological conditions, including stroke. 5 10 11 It includes 13 daily tasks, such as standing up from a chair, stepping onto a platform, or entering the shower, scored across three integrated domains: Functional Ability, Equipment Use (orthosis and assistive devices), and Performance Time. This multidimensional structure enables the LE-MFT to provide a detailed profile of lower-limb function by combining measures of task quality, the use of compensatory strategies (such as devices), and time efficiency.

Unlike other mobility measures, the LE-MFT assesses how the individual performs each task in a real-life setting, with or without adaptations, enabling clinicians to detect subtle improvements in function and independence. Its design supports clinical decision-making and progress tracking by better capturing the complexity of lower-limb motor recovery compared to gait speed or timed tests alone. 12

Although the LE-MFT has demonstrated clinical usefulness in North American settings, 10 11 no culturally-adapted version is currently available for use in Brazil. The development of a validated Brazilian Portuguese version could enhance clinical and research assessments by providing a standardized tool sensitive to different dimensions of functional mobility in people with chronic stroke.

Therefore, the objective of the current study was to translate and cross-culturally adapt the LE-MFT into Brazilian Portuguese and to evaluate its measurement properties, including interrater reliability, internal consistency, construct validity, and floor and ceiling effects in individuals with chronic stroke.

## METHODS

The current was a methodological cross-sectional study. To assess interrater reliability, data were collected through independent and concurrent evaluations performed by multiple raters. No test–retest design was performed. This study followed the Consensus-Based Standards for the Selection of Health Measurement Instruments (COSMIN) 13 14 and the Guidelines for Reporting Reliability and Agreement Studies (GRRAS). 15 The aim was to translate, culturally adapt, and evaluate the measurement properties of the Brazilian Portuguese version of the LE-MFT in adults with chronic hemiparesis. The measurement properties assessed included interrater reliability, internal consistency, construct validity, and floor and ceiling effects. The study was approved by the Ethics in Research Committee of Universidade Federal de São Carlos (under protocol number 3.710.548), and all participants provided written informed consent prior to enrollment.

### Description of the LE-MFT

The LE-MFT is a standardized observational instrument designed to assess functional motor performance of the lower limbs in adults with neurological conditions, such as stroke. 16 The test includes 16 functional tasks, which involve key lower-limb abilities commonly required for daily mobility and balance. The items are adapted from established clinical tests such as the Berg Balance Scale, the Functional Reach Test (Duke Mobility Skills Profile), and the Dynamic Gait Index. Each item is rated using three independent scales:

- Functional Ability Scale (FAS): It evaluates the quality of movement, including speed, coordination, and compensatory strategies, on a 0-to-10 scale, in which 10 represents normal movement and 0, inability to complete the task.
- Performance Time Scale: It records the time in seconds required to complete the task, with lower values indicating better performance.
- Equipment Scale: It assesses the type of assistance required, including orthotic devices or assistive mobility aids.

The score ranges from 0 (task completed only with major aid) to 10 (task completed independently, without aid). It is the average of two subscores: one for orthoses and one for assistive devices.

An Adjusted Functional Ability score can be calculated by averaging the FAS and Equipment Scale scores for each item, providing a more comprehensive representation of functional independence. Similarly, an Adjusted Performance Time Scale score is calculated by dividing the time taken to complete each task by its adjusted FAS score.

Fifteen of the 16 items are timed, with the exception of “Stand Forward & Reach,” which is used to assess stability and reach but is not timed. The test is administered by a trained clinician and typically video-recorded to enable accurate and blinded scoring. The LE-MFT enables a comprehensive assessment of lower-limb function, considering not only task performance but also movement quality and reliance on external aids.

Unlike traditional lower-limb assessments that focus solely on time or task completion, the LE-MFT incorporates three key dimensions: performance speed, movement quality, and the level of assistance required. This enables clinicians and researchers to detect subtle variations in motor function, especially in individuals who rely on orthotics or assistive devices. Its ability to quantify both capacity and real-world functional performance makes the LE-MFT particularly suited for use in rehabilitation trials and clinical monitoring of stroke-related motor impairments. The full list of LE-MFT tasks and scoring criteria in their original and translated forms is provided in the **Supplementary Material** (available at https://www.arquivosdeneuropsiquiatria.org/wp-content/uploads/2026/04/ANP-2025.0397-Supplementary-Material.docx ).

### Translation and cross-cultural adaptation

The translation and cross-cultural adaptation of the LE-MFT manual and data collection form followed Beaton's guidelines, 17 using the forward–backward translation method combined with expert panel review. This process aimed to ensure semantic, idiomatic, conceptual, and experiential equivalence between the original and Brazilian Portuguese versions. The authors of the original LE-MFT were contacted and approved the cross-cultural adaptation. Two of the original authors participated as coauthors of the present study. The translation process included five stages:

Stage 1: Initially, two independent translators, both native speakers of Brazilian Portuguese and fluent in English, produced separate forward translations into Brazilian Portuguese. One translator had clinical experience with the concepts evaluated by the instrument, while the other had no background in health sciences.

Stage 2: The translations were compared and synthesized by an expert panel comprising two physical therapists and one occupational therapist, all of whom had clinical and academic expertise in neurological rehabilitation. The panel analyzed discrepancies and agreed on a preliminary Portuguese version of LE-MFT. Consensus was reached through structured item-by-item comparison of the forward translations.

Stage 3: During pretesting, 10 physical therapists administered the prefinal version to 50 stroke survivors. The therapists documented participants' comprehension, any need for clarification, and observations regarding item execution. Verbal feedback from the participants was also collected regarding clarity and cultural relevance. The expert panel reviewed and categorized the feedback, which guided minor refinements in wording and instructions. Consensus was reached again after reviewing the pretesting feedback and implementing minor adjustments. Although pretesting was conducted to ensure clarity, cultural relevance, and acceptability of the translated items, the procedures were not intended to constitute a structured content-validity assessment as defined by COSMIN. No formal criteria nor quantitative indices (such as the Content Validity Index, CVI) were used to evaluate item relevance or comprehensiveness. Feedback from clinicians and participants was collected qualitatively and used for minor revisions only.

Stage 4: Backward translation was performed by two bilingual translators who were blinded to the objectives of the study and had no medical training. One of the translators was a native English speaker.

Stage 5: The expert panel reviewed the back-translations in comparison with the original instrument and confirmed equivalence. No significant semantic or cultural discrepancies were identified between the original LEMFT and the Brazilian version. A committee jointly confirmed equivalence regarding the back-translations and the original instrument.

### Measurement properties

#### *Construct validity*

Construct validity was examined by testing whether LE-MFT Brazil scores correlated with two established mobility performance measures: the 10MWT and the TUG test. These mobility instruments were selected based on strong evidence supporting their suitability to evaluate mobility outcomes in individuals with chronic stroke 6 18 19 Based on the literature, we predefined the expected direction and magnitude of these relationships. We hypothesized that LE-MFT Brazil scores would show a moderate-to-strong positive correlation with self-selected walking speed on the 10MWT, and a moderate-to-strong negative correlation with the performance on the TUG test. 6 18

#### *Reliability*

To establish the interrater reliability, internal consistency, and standard error of measurement of the LE-MFT Brazil, 2 physical therapists independently analyzed video recordings of the 50 participants with chronic stroke performing the test. The scores were subsequently compared. One of the raters received formal training in the original version of the scale at the University of Alabama at Birmingham, while the other used only the translated manual. Both were blinded to each other's assessments. High agreement between raters supports the consistency of the instrument when used by different clinicians.

#### *Floor and ceiling effects*

Floor and ceiling effects were examined for each LE-MFT scale by calculating the percentage of participants who obtained the lowest (0) or highest (10) possible score. According to standard psychometric criteria, the presence of floor or ceiling effects was defined as more than 15% of participants scoring at the extremes. 19 The analysis aimed to determine whether the LE-MFT Brazil was capable of capturing the full range of lower-limb function in individuals with chronic stroke.

### Participants

A convenience sample of 50 adults with chronic hemiparesis (≥6 months poststroke) was recruited from the community and through referrals from physical therapists working in rehabilitation services. The sample size was determined a priori based on the COSMIN guidelines, which recommend a minimum of 50 participants for studies evaluating measurement properties of measurement instruments. 13 The participants met the following inclusion criteria: diagnosis of chronic stroke, ability to ambulate at least household distances, and cognitive ability to follow instructions. The exclusion criteria were comorbidities affecting gait or inability to complete the assessment. 20

### Statistical analysis

Participant characteristics were summarized using descriptive statistics, including means, standard deviations (SDs), frequencies, and percentages. Data distribution was assessed using the Shapiro–Wilk test and visual inspection of histograms to verify assumptions of normality.

The interrater reliability of the LE-MFT Brazil was assessed using intraclass correlation coefficients (ICCs) for each scoring domain. ICCs were estimated using a two-way random-effects model with absolute agreement for single measurements [ICC(2,1)].as both raters were considered representative of a larger population of evaluators. Values of the ICC ≥ 0.70 were interpreted as acceptable, 21 values ≥ 0.80, as good, and values ≥ 0.90, as excellent reliability.

Measurement error was quantified by calculating the standard error of measurement (SEM) using the formula SEM = SD × √(1 - ICC). Based on the SEM, the smallest real difference (SRD) at the individual level was calculated as SRD = 1.96 × SEM × √2, representing the minimum change required to be interpreted as a true change beyond measurement error. 22

Interrater agreement was further examined using Bland–Altman analyses for the FAS and Performance Time scores. Mean differences (bias) and 95% limits of agreement were calculated to identify systematic differences between raters and to assess the magnitude and distribution of the measurement error across the range of scores. The presence of proportional bias was visually inspected by examining the dispersion of differences relative to the mean values.

Internal consistency was assessed using Cronbach's α coefficients calculated across the 16 test items for each LE-MFT scoring domain, including the FAS, the Equipment Scale, the Adjusted Functional Ability Score, and the Performance Time Scale. Cronbach's α values ≥ 0.70 were considered indicative of adequate internal consistency. 23 Item-deletion analyses were conducted to determine whether removal of any individual item resulted in substantial changes in α values. 23

Construct validity was evaluated by testing predefined hypotheses regarding the relationships involving LE-MFT Brazil scores and established measures of mobility performance. Pearson's correlation coefficients were calculated between the Adjusted Functional Ability Score and Performance Time Score of the LE-MFT and the TUG test and the 10MWT. Correlation magnitudes were interpreted as excellent (≥ 0.75), moderate (0.50–0.74), fair (0.25–0.49), or low (< 0.25). 24 Ninety-five percent CIs were calculated for all correlation coefficients to provide estimates of precision. Statistical significance was set at *p* < 0.05.

Floor and ceiling effects were examined for each LE-MFT scale by calculating the percentage of participants who achieved the minimum or maximum possible scores. Floor or ceiling effects were considered present if more than 15% of the participants scored at the extremes.

All statistical analyses were performed using the Statistical Package for the Social Sciences, version 19.0 (SPSS Inc., Chicago, Illinois) software, and no missing outcome data were observed; therefore, no data imputation procedures were required.

## RESULTS

### Participant characteristics

In total, 83 individuals were screened, 50 of whom met the inclusion criteria and completed the evaluation. The exclusions were mainly related to non-compliance with the cognitive eligibility criteria. There were no postenrollment exclusions or missing data. A total of 50 adults with chronic stroke (30 men and 20 women) participated in the study. The mean age was of 63 ± 13 (range = 35–86) years, and the mean time since stroke onset was of 54 ± 58.4 (range = 7–114) months. Half of the participants had motor impairment resulting from a stroke in the left hemisphere, and 40% had paresis of the dominant lower limb. Regarding mobility assistance, 58% of the participants did not use any assistive devices during the assessment, while 42% used one or more walking aid devices. Among device users, canes were the most frequently used ( *n* = 15), followed by crutches ( *n* = 5) and a standard walker without wheels ( *n* = 1); Some participants used lower limb orthoses (such as ankle-foot orthoses [AFO]/ knee-ankle-foot orthoses [KAFO]) in combination with walking aids. According to the classification proposed by Lin et al., 9 59.5% of the participants were categorized as community walkers, 23.8%, as limited community walkers, and 16.7%, as home walkers.

### Translation and cross-cultural adaptation

The cross-cultural adaptation of the LE-MFT followed international guidelines for patient-reported outcome measures and observational tools, including the five-step process of translation, synthesis, back-translation, expert review, and pretesting. 16 Feedback obtained during pretesting indicated that all items were generally well understood, requiring only minor refinements. However, because no formal content validity metrics were applied, these findings should be interpreted as qualitative insights rather than quantitative evidence of content validity. The original manual and data collection form were first translated into Brazilian Portuguese by two independent translators, and discrepancies were resolved by consensus.

During the expert panel review, several semantic and formatting adjustments were made to ensure conceptual equivalence and clinical applicability. Verbs originally written in the infinitive form (such as *to support* , *to encourage* ), for example, were converted to the present tense ( *supports* , *encourages* ) to provide clearer instructions consistent with common phrasing in clinical protocols. The phrase *affected side* was standardized using the abbreviations E ( *esquerdo* , ‘left’ in Portuguese) and D ( *direito* , ‘right’ in Portuguese), enabling a consistent identification of laterality across items and reducing ambiguity in scoring.

Instructions were restructured to follow a more directive tone, and minor rewordings were introduced to simplify the sentence structure without altering the original meaning. The response scales and observation forms were reformatted for improved legibility and usability in practice. Specifically, new fields were included to identify the evaluator, document the date of assessment, and register the phase of treatment, aligning with Brazilian clinical documentation practices.

These adjustments were approved by the original authors of the LE-MFT and incorporated into the final version of the Brazilian Portuguese instrument. A summary of the key adaptations is provided in Tables 1 2 3 4 .

**Table 1:** Functional tasks

Original Item (English)	Translated term (Portuguese)	Cross-cultural adaptation	Comments
Sit to stand	*Passar de sentado para de pé*	Semantic adjustment	Expression chosen to sound natural in Brazilian Portuguese clinical use
Stand/Forward reach	*Em pé/Alcance anterior*	Semantic simplification	Maintained meaning, simplified wording for clarity
Turn 360° to right	*Girar 360° para a direita*	Literal translation	No cultural issues
Turn 360° to left	*Girar 360° para a esquerda*	Literal translation	No cultural issues
Place right foot on stool	*Colocar o pé direito em um banco*	‘Stool’ → *banco*	Chosen as more familiar term in Brazil
Place left foot on stool	*Colocar o pé esquerdo em um banco*	Same as above	Maintains equivalence
Place right foot on stool with 5-lb (2.3-kg) weight	*Colocar o pé direito em um banco com peso (de 2,3 kg)*	Adapted unit	Pound converted to kg for the Brazilian context
Place left foot on stool with 5-lb (2.3-kg) weight	*Colocar o pé esquerdo em um banco com peso (de 2,3 kg)*	Adapted unit	Same as above
Standing/Abduction slide to right	*Em pé / abdução da perna para a direita*	Literal translation	Maintains clinical terminology
Standing/Abduction slide to left	*Em pé/Abdução da perna para a esquerda*	Literal translation	Maintains clinical terminology
Step over shoeboxes	*Passar sobre caixas de sapato*	Object adaptation	‘Shoebox’ is not common; clarified as *caixas* for the Brazilian context
Retrieve the object from the floor	*Pegar objeto do chão*	Semantic simplification	Simplified for better comprehension
Look over your shoulder while walking	*Olhar sobre os ombros enquanto anda*	Literal translation	Maintains meaning, natural in Portuguese
Step around cones	*Contornar cones*	Simplification	Clear and commonly used in rehabilitation
Step up one flight of stairs	*Subir um lance de escadas*	Literal translation	Maintains meaning
Step down one flight of stairs	*Descer um lance de escadas*	Literal translation	Maintains meaning
Walk 10 meters	*Caminhar 10 metros*	Unit adapted	Converted from feet to meters

**Table 2:** Functional Ability Scale

Original (English)	Translation (Portuguese)	Adaptation	Comment
10–Able to stand independently, no hands on the chair, stabilizes independently	*10–Capaz de ficar em pé de forma independente, sem apoio das mãos na cadeira, estabiliza-se sozinho*	Literal	Clear equivalence
9–Able to stand independently, no hands, more than one try needed	*9–Capaz de ficar em pé de forma independente, sem apoio, necessitando de mais de uma tentativa*	Semantic simplification	Adapted wording
7–Able to stand independently using one hand on chair	*7–Capaz de ficar em pé de forma independente usando uma mão na cadeira*	Literal	Maintains meaning
6–Able to stand independently using both hands	*6–Capaz de ficar em pé de forma independente usando as duas mãos*	Literal	Maintains meaning
5–Able to stand independently but requiring more than one try	*5–Capaz de ficar em pé de forma independente, mas necessitando de mais de uma tentativa*	Simplification	Natural Portuguese
3–Able to stand with contact-guard assistance	*3–Capaz de ficar em pé com auxílio de contato*	Semantic adaptation	‘Contact-guard’ adapted to Portuguese terminology
1–Able to stand with minimal to maximal assistance	*1–Capaz de ficar em pé com assistência mínima a máxima*	Literal	Maintains range
0–Cannot stand or requires assistance of two or more persons	*0–Incapaz de ficar em pé ou necessita de assistência de duas ou mais pessoas*	Literal	Equivalent

**Table 3:** Orthotic Scale

Original (English)	Translation (Portuguese)	Adaptation	Comment
5–No orthosis	*5–Sem órtese*	Literal	No issues
4–Shoe insert	*4–Palmilha*	Cultural adaptation	‘Shoe insert’: *palmilha*
3–Ankle-foot orthosis (AFO)	*3–Órtese tornozelo-pé (AFO)*	Literal + acronym	Kept the AFO acronym
2–Knee-ankle-foot orthosis (KAFO)	*2–Órtese joelho-tornozelo-pé (KAFO)*	Literal + acronym	Standard term
1–Hip-knee-ankle-foot orthosis (HKAFO)	*1–Órtese quadril-joelho-tornozelo-pé (HKAFO)*	Literal + acronym	Standard term
0–Other/Not specified	*0–Outra/Não especificada*	Literal	Maintains equivalence

**Table 4:** Assistive Device Scale

Original (English)	Translation (Portuguese)	Adaptation	Comment
5–No assistive device	*5–Sem dispositivo auxiliar*	Literal	No issues
4–Cane (single-point)	*4–Bengala (simples)*	Semantic adaptation	Clear Brazilian usage
3–Quad cane	*3–Bengala de quatro apoios*	Semantic adaptation	Adjusted to local terminology
2–Crutches	*2–Muletas*	Literal	Common term
1–Walker	*1–Andador*	Literal	Common clinical term
0–Wheelchair	*0–Cadeira de rodas*	Literal	No issues

### Construct validity

**Figure 1:**
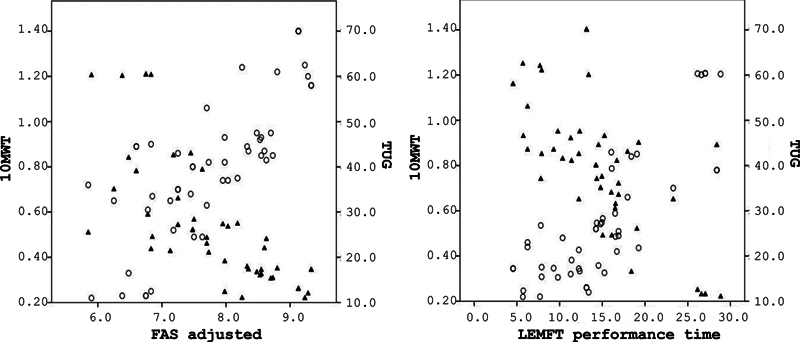
Abbreviations: LE-MFT, Lower Extremity Motor Function Test; FAS, Functional Ability Scale; TUG, Timed Up and Go; 10MWT, 10-Meter Walk Test. Notes: ▲ for the TUG test and ○ for the 10MWT. Adjusted Functional Ability and Performance Time Scores on the LE-MFT versus 10MWT ang TUG test scores.

Construct validity was examined by correlating the LE-MFT Brazil scores with established mobility measures. The adjusted FAS score showed a strong positive correlation with the 10MWT (r = 0.77; 95%CI: 0.65–0.87; *p* < 0.001) and a strong negative correlation with the TUG test (r = −0.75; 95%CI: −0.85 to −0.64; *p* < 0.001). Similarly, the Performance Time score showed a strong positive correlation with the TUG test (r = 0.76; 95%CI: 0.61–0.86; *p* < 0.001) and a moderate-to-strong negative correlation with the 10MWT (r = −0.65; 95%CI: −0.80 to −0.45; *p* < 0.001). All correlations were statistically significant, in the expected direction and of moderate-to-strong magnitude, providing robust support for the instrument's construct validity. All correlation coefficients are presented with their corresponding 95%CIs to improve interpretability and assess the precision of the estimates ( Figure 1 ).

### Reliability

Cronbach's α coefficients indicated excellent internal consistency for all LE-MFT scales, with values of 0.96 for the FAS, 0.86 for the Equipment Scale, 0.95 for the Adjusted FAS, and 0.93 for the Performance Time Scale, all exceeding the recommended threshold of 0.70. The item exclusion analyses showed stable internal consistency in all items, with Cronbach's α values ranging from 0.95 to 0.98 for the FAS, from 0.85 to 0.88 for the Equipment Scale, from 0.93 to 0.96 for the Adjusted FAS, and from 0.91 to 0.95 for the Performance Time Scale. The removal of any individual item did not result in a significant increase in α values, indicating that all items contributed adequately to the scales and that no item redundancy was identified. The SEM and minimal detectable change at the 95% confidence level (MDC95) values for all LE-MFT domains are presented in Table 5 . At the individual level, the minimum change required to exceed measurement error was of 3.74 seconds for Performance Time, 0.94 points for the Equipment Scale, 1.14 points for the FAS, and 1.00 point for the Adjusted FAS. These values provide clinically-useful thresholds to interpret change over time. These results indicate the minimum amount of change required to exceed measurement error and be interpreted as a true change in performance.

**Table 5:** Reproducibility of the LE-MFT Brazil ( *n* = 50)

	Cronbach's α coefficient	Floor effect (n)	Ceiling effect (n)	ICC (95%Cl)	Standard error of measurement (SEM)	MDC95
Performance Time Scale (seconds)	0.95	0	0	0.96 (0.87–0.96)	1.35	3.74
Equipment Scale (0–10)	0.94	0	19	0.87 (0.77–0.93)	0.34	0.94
FAS (0–10)	0.92	0	0	0.93 (0.87–0.96)	0.41	1.14
Adjusted FAS (0–10)	0.90	0	0	0.86 (0.75–0.92)	0.36	1.00

Abbreviations: FAS, Functional Ability Scale; ICC, intraclass correlation coefficient; LE-MFT, Lower Extremity Motor Function Test; MDC95, minimal detectable change at the 95% confidence level.

**Figure 2:**
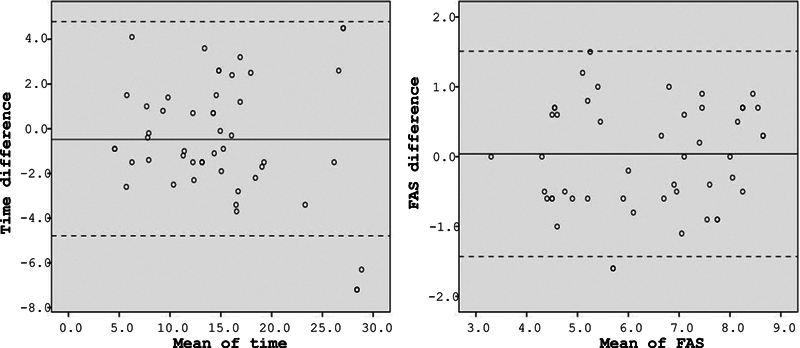
Interrater agreement regarding the scores on the Functional Ability Scale (FAS) and Performance Time Scale.

The interrater agreement was further examined using Bland–Altman analyses for the scores on the FAS and the Performance Time Scale. The plots illustrate minimal systematic bias between raters and show that most observations fall within the 95% limits of agreement, supporting good agreement between evaluators ( Figure 2 ).

## DISCUSSION

The findings of the current study support the use of the Brazilian Portuguese version of LE-MFT as a culturally-appropriate and psychometrically-sound instrument to assess lower-limb motor performance in individuals with chronic stroke. The forward–backward translation process and expert panel review preserved the conceptual integrity of the original version, ensuring semantic equivalence and clarity for Brazilian users. No inconsistencies were found during pretesting, and the final version was approved by the original developers.

The LE-MFT Brazil demonstrated excellent interrater reliability, with ICC values ranging from 0.87 to 0.99 across all domains. Internal consistency was also high, with Cronbach's α coefficients above 0.85 in all scoring domains. These values meet the standards recommended for reliability studies and indicate that the instrument provides consistent scoring across different raters. 11 17 25 The SEM and MDC95 values improve the clinical interpretability of the LE-MFT Brazil by providing objective thresholds to distinguish true change from measurement error. At the individual level, a person with chronic stroke would need to improve by at least 3.74 seconds on the score on the Performance Time Scale, 1.14 points on the FAS, and 1.00 point on the Adjusted FAS for the observed change to be interpreted as a real functional gain rather than measurement noise. For the Equipment Scale, a change of ∼ 0.94 points would be required. These values may help clinicians and researchers interpret longitudinal changes more confidently when monitoring recovery or evaluating intervention effects in individuals with chronic stroke. The measurement properties observed in the LE-MFT Brazil were generally consistent with those reported in the original instrument, particularly regarding internal consistency and interrater reliability. Differences in equipment-related scores may reflect functional characteristics of the Brazilian sample. To date, no other international adaptations have been published, limiting broader cross-cultural comparison.

Construct validity was supported by moderate-to-strong correlations involving the LE-MFT Brazil and established mobility assessments, including the TUG test and the 10MWT, both widely used and validated in stroke populations. 9 26 These associations reinforce the ability of the LE-MFT to capture meaningful variations in lower-limb motor performance. Beyond these findings, the LE-MFT Brazil offers added clinical value by providing an integrated assessment that incorporates movement quality, the use of assistive devices, and task-specific performance, elements not captured by time- or distance-based measures such as the TUG test and 10MWT. This multidimensional approach supports more precise clinical reasoning and individualized rehabilitation planning. However, because the TUG test and the 10MWT do not encompass the full spectrum of lower-limb motor function assessed by the LE-MFT, future studies should expand construct-validity testing by including complementary measures, such as the Fugl-Meyer Assessment of Lower Extremity and standardized balance assessments (such as the Berg Balance Scale or Dynamic Gait Index).

The Equipment Scale domain showed a ceiling effect, with most participants (62%) not using assistive devices, likely reflecting the relatively-high independence level of this sample rather than a limitation of the scale. The functional homogeneity of our sample, composed predominantly of community ambulators, contributed to the ceiling effect observed in the Equipment subscale and may limit the generalizability of our findings to individuals with more severe gait impairments. However, this profile is consistent with previous cohorts of community-dwelling individuals with chronic stroke, in which assistive-device use was relatively uncommon, partly because some individuals had discontinued device use or no longer required it. 25 27 Future studies should recruit individuals with a wider range of motor impairments and greater reliance on assistive devices to enhance external validity and reduce ceiling effects.

In addition to these sample-related limitations, the current study provides an initial evaluation of the measurement properties of the LE-MFT Brazil. However, several key psychometric domains, including formal content validity, test–retest reliability, and responsiveness were not assessed. Although interrater measurement error was examined through SEM and MDC95 estimates, temporal measurement stability and responsiveness still need to be established in future studies. Although excellent interrater reliability was observed, the temporal stability of the LE-MFT Brazil could not be determined, underscoring the need for future studies to incorporate a test–retest design with an appropriate retesting interval to evaluate score reproducibility over time.

Furthermore, a key limitation is the absence of a structured assessment of content validity following COSMIN standards. Although pretesting supported the clarity and cultural relevance of the translated items, no formal criteria were applied to evaluate item relevance, comprehensiveness, or clarity, nor were quantitative methods, such as the CVI, used. Therefore, future studies should incorporate a systematic content-validity evaluation involving a multidisciplinary expert panel and end-user perspectives (such as clinicians and individuals poststroke), supported by quantitative agreement metrics.

Overall, the LE-MFT Brazil is a comprehensive and clinically-relevant tool, capable of capturing the complexity of lower-limb functional performance in adults with chronic hemiparesis. Its structure enables a detailed assessment of task performance and adaptations, offering a valuable resource for rehabilitation planning and monitoring.

In conclusion, the Brazilian Portuguese version of the LE-MFT showed strong measurement properties, including excellent interrater reliability, high internal consistency, and construct validity when used with individuals who have chronic stroke. The multidimensional scoring system covering functional ability, equipment uses, and performance time offers a comprehensive and ecologically-valid assessment of lower-extremity motor function. A major strength of the LE-MFT is its ability to capture performance quality and the effect of assistive strategies on task execution, setting it apart from commonly-used gait and mobility tests. Scoring based on video recordings was dependable and may provide a practical alternative in clinical and research settings when real-time scoring is not feasible. The LE-MFT Brazil is a valuable addition to the tools available to assess functional mobility and guide rehabilitation in adults with hemiparesis after stroke.
